# Cost Analysis of Integrative Inpatient Treatment Based on DRG Data: The Example of Anthroposophic Medicine

**DOI:** 10.1155/2013/748932

**Published:** 2013-01-31

**Authors:** Jürgen Heinz, Wolfgang Fiori, Peter Heusser, Thomas Ostermann

**Affiliations:** ^1^Öschelbronn Clinic, Center for Integrative Medicine, Am Eichhof 30, 75223 Niefern-Öschelbronn, Germany; ^2^DRG Research Group, University Hospital Münster, 48129 Münster, Germany; ^3^Center for Integrative Medicine, University of Witten/Herdecke, Gerhard-Kienle-Weg 4, 58313 Herdecke, Germany

## Abstract

*Background.* Much work has been done to evaluate the outcome of integrative inpatient treatment but scarcely the costs. This paper evaluates the costs for inpatient treatment in three anthroposophic hospitals (AHs). *Material and Methods.* Cost and performance data from a total of 23,180 cases were analyzed and compared to national reference data. Subgroup analysis was performed between the cases with and without anthroposophic medical complex (AMC) treatment. *Results.* Costs and length of stay in the cases without AMC displayed no relevant differences compared to the national reference data. In contrast the inlier cases with AMC caused an average of € 1,394 more costs. However costs per diem were not higher than those in the national reference data. Hence, the delivery of AMC was associated with a prolonged length of stay. 46.6% of the cases with AMC were high outliers. Only 10.6% of the inlier cases with AMC were discharged before reaching the mean length of stay of each DRG. *Discussion.* Treatment in an AH is not generally associated with an increased use of resources. However, the provision of AMC leads to a prolonged length of stay and cannot be adequately reimbursed by the current G-DRG system. Due to the heterogeneity of the patient population, an additional payment should be negotiated individually.

## 1. Introduction

Integrative medicine according the US National Center for Complementary and Alternative Medicine “combines treatments from conventional medicine and complementary medicine (CAM) for which there is some high-quality evidence of safety and effectiveness” [1]. One of the approaches which fits this definition is anthroposophic medicine (AM) established in the 1920s by Rudolf Steiner and Ita Wegman along with some other doctors [2]. In the last decades, AM has developed to become one of the main representatives of integrative medicine in Germany and is currently practised in over 60 countries [3].

Furthermore, it is explicitly designated and mentioned in the German Drug Law as a “Special Therapeutic System” alongside herbal medicine and homeopathy [4]. AM explicitly sees itself as an extension and supplement to the “conventional medicine” and not as an alternative medicine [5]. A special feature of AM compared to other integrative medical disciplines is that AM is established both in the outpatient sector and in many acute hospitals [6].

Since 2002 German hospital payment is based on the German refined diagnosis-related groups (G-DRGs) [7]. DRGs are defined by the patients' diagnoses, gender and age, treatment procedures, complications or comorbidities, and further attributes. Based on this data, a predetermined rate per case is calculated.

The German official classification of operational procedures (*Operationen und Prozedurenschlüssel*: OPS) is used to code operations and other medical procedures. Within the OPS, anthroposophic medical complex treatment was established in 2005 as a special code (OPS 8-975.3; Table 1) [8, 9]. This is owed to the fact that AM requires an intense use of resources (counseling, diagnosis, and treatment planning), therapeutic intervention (physical therapy, such as eurhythmy, art therapy, music therapy, and rhythmical massage), and nursing interventions (external applications such as wound and liniments). Once a patient receives at least 30 therapeutic units within his inpatient treatment coded with this digit, an additional unweighted payment “ZE-26” (anthroposophic complex medical treatment additional payment) is generated [9, 10]. This additional payment has to be negotiated and agreed on by each hospital individually with insurance companies as a part of the remuneration negotiations according to the hospital remuneration act [11]. The compensation currently varies from hospital to hospital. 

The present study examined whether the provision of AMC is associated with an increased use of resources.

## 2. Material and Methods

### 2.1. Data Selection

**Figure 1:**
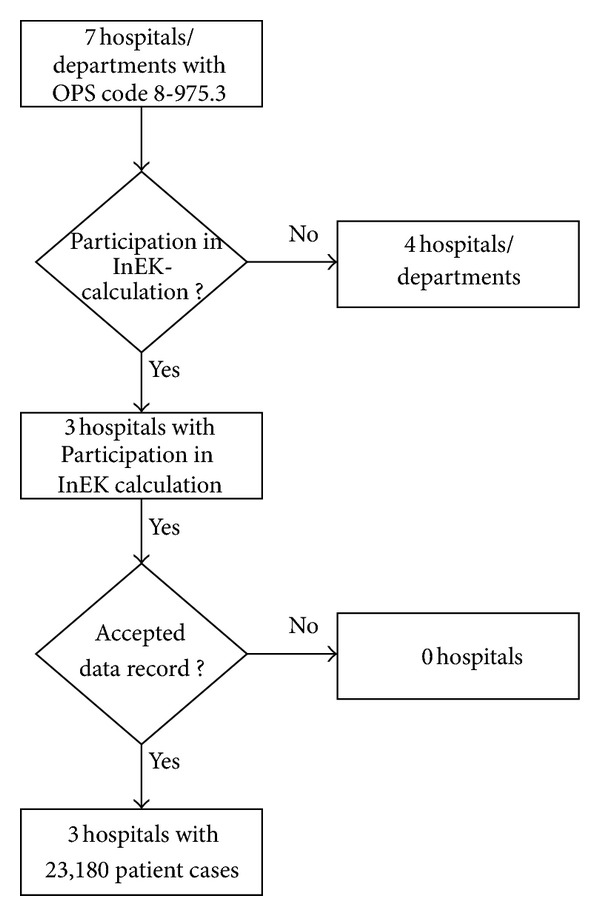
Flow chart of the selection process of hospitals.

The current analysis is based on German cost and performance data from 2009 which had already been approved and sent to the Institute for the Hospital Remuneration System (InEK). From the seven AM hospitals or departments which calculate the ZE-26 in Germany, three hospitals, namely, the Community hospital Havelhöhe (Berlin), the Community hospital Herdecke, and the hospital Öschelbronn, take part in the annual cost calculation of InEK. Thus, valid cost data are only available from these three institutions (Figure 1). These data were grouped into the 2011 version of the G-DRG system and valued at the federal base rate 2011. 

Weighted additional payment was considered in compliance with the price of the Case Fees Agreement (German: Fallpauschalenvereinbarung FPV) of 2011. Unweighted additional payments were, if possible, taken into account using the individual hospital prices from 2009. In the rare cases in which no individual hospital arrangement was made, treatments were considered with 600€. Cases which were grouped into unweighted G-DRGs were already subject to the individual hospital agreement anyway and were not included in the analysis. The length of stay was always calculated in days of occupancy, so the day of discharge was not considered unless it was also the day of admission.

**Figure 2:**
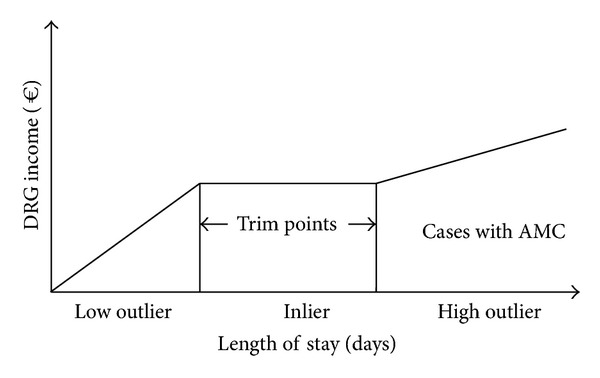
Length of stay: definition of inlier and outlier cases (annually calculated for each DRG individually).

Cases were separated into two groups: those with anthroposophic medical complex (AMC) treatment and those without. Collected data was compared between the included hospitals and the with national reference data published by the German Institute for the Hospital Remuneration System (InEK). Cases with the length of stay between individually for each DRG defined boundaries (lower and upper trim point) are named “inliers.” Cases with a length of stay longer than the upper trim point of the DRG into grouped are referred to as high outliers, while cases that stay shorter than the lower trim point which they are referred to as low outliers, respectively (Figure 2). They are subject to special surcharges/deductions to their relative weight. Cases transferred to or transferred from other hospitals that stay shorter than the average length of stay of the respective DRG are also subject to deductions to their relative weight and not considered as inliers. As the reference group only includes inliers certain analyses could only be made with the respective cases of collected data. 

With costs deriving from 2009 and revenues calculated with the base rate from 2011, costs and revenues cannot be matched directly as, for example, the development of the base rate should partly compensate for rising costs.

### 2.2. Statistical Analysis

The average DRG costs of inlier cases of the reference group are based on a cost matrix published by the Institute for the Hospital Remuneration System (InEK). These costs do not include costs reimbursed by additional payments (ZE). To be able to compare the collected cost data with the InEK reference group, the costs of each inlier case were adjusted for included costs for additional payments by the amount of reimbursement the additional payments would have realized. In the DRG costing, a distinction is made within the InEK cost matrix for the determination of deductions and surcharges for outliers. While the costs for the “key service” are regarded relatively independent of length of stay, the so-called “differential costs” are considered to be dependent on the length of stay and therefore used to calculate deductions and surcharges. The “key service” is defined as the sum of the cost values in the account groups 04 (operating room), 05 (anesthesia), 06 (delivery room), 07 (cardiac diagnosis/treatment), and 08 (endoscopic diagnostics/therapy) plus the cost values of the cost element group 05 (implants) which have not yet been included. The remaining costs of the InEK cost matrix are referred to as “differential costs.”

Statistical analysis was performed with SPSS 18.0 for Windows. Descriptive analysis was used to determine rates and proportions. Means and standard deviations (SDs) were calculated for continuous data. Two-tailed Chi-square test was used to analyze differences in frequencies; *t*-test was used to analyze differences in means. A *P* value of less than 0.05 was regarded as indicating a statistically significant difference.

### 2.3. Ethical Considerations

The present study is based on secondary data collected from the hospitals and for the reference group provided by the InEK. As such, the recommendations for good practice in secondary data analysis (e.g., anonymization of data on prescriptions and diagnoses) developed among others by the German Working Group on the Collection and Use of Secondary Data were applied in full [12]. 

## 3. Results

### 3.1. Sample Description

From the three hospitals included in our analysis, a total of 23,180 cases discharged in 2009 were available. Of them, only 1,331 cases (6.1%) received AMC. This value varies considerably from hospital to hospital and ranges between 2.6 and 19.8% (Table 2; *P* < 0.001). 

The patient groups or types of diseases were also heterogeneous. The spread ranges over 308 different G-DRGs corresponding to 26% of all inpatient G-DRGs at a level of 1,189 in the 2011 version of the G-DRG system. An accumulation was found in the medical collectives of solid malignant neoplasm, chronic diseases such as heart failure, COPD/asthma, hypertension, diabetes mellitus, gastritis, inflammatory bowel disease, and psychosomatic or psychiatric principal diagnoses (Table 2).

The respective case collectives receiving AMC differ clearly among the three participating hospitals. The average DRG cost weight *CMI* (case mix index) was 2.16 among the cases with AMC; cases without this AMC achieved on average a CMI of only 0.83. Thus AMC cases achieve an average DRG income 2.6 times higher. The CMI indicates already higher resource consumption. 

Cases classified as “inliers” without AMC (*N* = 21,849) caused an average of InEK-compliant costs of 2,451€ (SD: 3,037€), while “inlier” cases with AMC (*N* = 1,331) amounted to 6,724€ (SD: 9,323€) which is significantly different (*t*-test, *P* < 0.001).

### 3.2. Comparison with a Reference Group

#### 3.2.1. Cases without AMC

The comparison of the average length of stay as well as the percentage of cases which stay shorter than the average residence times of the InEK reference collective (only inlier) shows no abnormalities for cases without AMC and also corresponds to the expected values (Table 3).

The adjusted costs of an inlier with an average of 2,394€ nearly correspond to the cost of 2,387€ stipulated by InEK (Table 3).

In the distribution of personnel, equipment, and infrastructure costs, as well as in the mean length of stay, no relevant differences to the InEK comparison group can be identified; thus treatment in anthroposophically oriented hospitals excluding AMC patients is not associated with an increased use of resources. 

#### 3.2.2. Cases with AMC

The average length of stay of cases with AMC was 19.5 days while the average in the inlier group was 14.7 days. In the InEK reference group (only inliers), the value for length of stay was given as 10.9 days which is 3.8 days shorter than the respective value of the AMC group. 

This is due to the fact that nearly half of the cases (46.6%) with AMC were high outliers while inliers amounted to 53.1%. Therefore low outliers only occurred in 0.3%. Only 5.9% of the cases were discharged before reaching the mean length of stay of each DRG. Moreover only 10.6% of the inliers with an AMC are discharged before reaching the mean length of stay of each DRG according to the DRG catalog.

In contrast to the cases without AMC, cost data of the cases with AMC was strikingly different. Cases with AMC produced average costs of 7,992€; inliers only produced adjusted costs of 6,638€ which was still 1,394€ higher than in the InEK reference group (5,244€). 

It is also noticeable that the cost of all cases, including the cost of the high percentage of high outliers (cases with longer stay than the upper trim point of the respective DRG) which are not published by the InEK, was again on average € 1,268€ higher than the not adjusted cost of the inliers, while the downstream revenues generated (without the additional payment for the AMC) were only 567€ on average. 

With respect to the “key service,” inliers with an AMC (1,078€) were on average only slightly more costly than cases of InEK calculation (1,009€). 

The striking differences between cases with/without AMC occurred in the differential costs (which are sensitive to the length of stay). It is remarkable that in the cases with AMC (inliers and high outliers) the differential costs per day were not higher than those in the InEK reference group. This shows that the additional costs of cases with an AMC are caused by a longer length of stay and not by the use of more resources per day.

## 4. Discussion

Much work has been spent to evaluate the outcome of integrative in-patient treatment [13, 14], but only some articles deal with the costs of such strategies [15, 16]. This article for the first time evaluates the costs for integrative in-patient treatment in three hospitals using cost and remuneration data, which in contrast to other approaches takes the perspective of the healthcare suppliers. This study in particular analyzes cases with anthroposophic medical complex treatment (AMC). 

As a first result, we found a wide dispersion of the G-DRG spectrum for the provision of AMC. This may be explained by the fact that the use of AM is patient-specific and, in addition to the clinical picture, determined by the personality of the patient and the patient's will [17]. Thus it is primarily not the type of illness or the DRG group which triggers the provision of the anthroposophic additional payment, but the individual therapeutic process negotiated between physician and patient. 

Our evaluation also revealed significant cost differences among the hospitals in the provision of AMC. Apart from patient physician interactions, this is owed to the hospital-specific processes and medical collectives treated. This in-house treatment structure was not a part of this evaluation but however should be analyzed in a subsequent study, in which, for example, by means of a clustering based on performance groups, the costs and treatments' side of comparable entities of the anthroposophic hospitals to be calculated are compared [18]. At this present time, it thus can be inferred that AMC should not be subject to a nationally standardized additional payment and should be determined and negotiated from hospital to hospital.

This hospital-specific procedure for the additional payment of the ZE-26 is not unique in the G-DRG system. According to the catalog for additional payments, there is a total of 64 payments listed which are to be negotiated individually with each hospital [10]. The majority of these are attributable to drugs, operational and medical interventions (e.g., *ZE2011-53* “additional charge stent graft prosthesis for aortic aneurysms with fenestration or branch”) which, due to their complexity or limited use, are not subject to the federal calculation. Still to be found in this catalog next to the anthroposophic complex medical treatment are three similar additional payments declared as “special treatments” by the InEK Institute, which, due to their characteristics, might be accompanied by a desired longer length of stay: the ZE2011-36 “care for the severely disabled,” the *ZE2011-40* “additional payment alternative complex treatment,” and the nationally weighted ZE60 “palliative complex treatment”. Whilst the literature research did not show any further useful information for the first two payments, it turns out that the additional payment for palliative care is affected by similar remuneration problems which are currently discussed in the literature [19] and lead to a calculated additional payment compensating for the desired prolonged length of stay [20]. 

Another study of Romeyke and Stummer [21] analyzed complex rheumatic treatment and similar to our study found a prolonged stay of patients without higher costs per day associated with this form of treatment. However that study used data from the beginning of the DRG calculation and reliability data was weak. Because of the limited range of DRGs affected, a specific DRG (I97Z) for the complex rheumatic treatment could be established and calculated meanwhile. Our study showed that the provision of AMC is associated with a prolonged length of stay, and this is not just since there is a compensation by the additional fee “ZE-26.” Thus, it is not the doctor or therapist input per day which leads to the financial shortfall compared to the InEK patient population with regard to the G-DRG remuneration, but rather the extended hospital stay which is not compensated by a high outlier surcharge which only partially compensates for an increased use of resources after the upper trim point of the DRG has been exceeded [22]. For inlier collectives with systematically longer stays that do not reach the upper trim point, no compensation exists at all.

Consequently all inpatients with a longer stay than the DRG-average tend to be remunerated with a deficit and not only the affected cases in anthroposophic hospitals. What is relevant, however, is that it can be balanced out, from an economic aspect, by cases with a short stay. After 55.7% of all cases and 50.7% of all inliers without AMC could be discharged before reaching the mean residence time of each DRG, there is no underfunding due to the length of stay for this case collectives. If, however, a longer residence is not due to inefficiency but to a medical specialty or complex treatment, such as the anthroposophic complex medical treatment, then a sanctioning of the economic disadvantages by a “right shift” of the lengths of stay (for long inliers and high outliers) is not appropriate.

## 5. Limitations

Although this study has used validated data, it still has limitations. Firstly 2,100 hospitals do exist in Germany, but only 113 of them take part in the annual cost calculation of InEK of which three are anthroposophic hospitals. This of course denotes an overproportional participation of anthroposophic hospitals in the annual calculation of InEK. But although the participation rate of anthroposophic hospitals is significantly higher than those of conventional hospitals, the large data basis of 4.5 million patients in total suggests that the bias caused by anthroposophic hospitals is marginal. 

Secondly no information about the most relevant diagnostic groups (represented by the MDC class in the DRG classification) is given. This is due to the fact that the cases with AMC distribute over a total of 308 G-DRGs and thus our data did not allow a valid clustering on diagnostic groups. As a consequence, we were not able to compare cases with or without AMC in more detail at the level of DRGs. This unfortunately leads to the problem of a certain amount of impreciseness while mapping difference costs and cases. However, this does not afflict the general results of this work.

Finally from a methodological point of view, cost data are notoriously skewed, which in our data is suggested by the high standard deviations relative to the means. Thus, other statistical approaches like bootstrapping might be more appropriate for this situation [23]. However, the structure of the raw data given for this analysis did not allow for more complex statistical tests.

## 6. Implications for Health Policy

Anthroposophic medicine in Germany is legally recognized as a special type of integrative treatment which is highly demanded on the patients' side. We were able to demonstrate that anthroposophic medicine at the moment can only establish itself in the acute in-patient sector when compensation of the increased use of resources calculated individually by each hospital is effected over the ZE-26.

Currently the compensation rates, at least among the three participating hospitals, do not cover the costs and are thus associated with a negative contribution margin per supplied AMC. Therefore the hospitals do not have any economical incentive to provide this type of medicine for economic reasons [24]. 

Whether anthroposophic medicine with its special use in therapy can establish itself under these conditions in an in-patient setting either midterm or long term, will mainly be a political or social decision which, in the end, should be supported by arguments as have been described and carried out in this paper.

## Figures and Tables

**Table 1:** OPS complex code 8-975.3 from OPS catalog 2011.

*8-975.3 anthroposophic medical complex treatment *	
The treatment is carried out using several specific therapies with a total of at least 30 therapy sessions (each of at least 30 minutes) from the following areas	
(i) Applications and baths	
(ii) Massages, rubs, and wraps	
(iii) Movement therapies	
(iv) Arts therapies	
(v) Supportive therapy and patient education	

**Table 2:** Patient allocation according to diagnoses and procedures.

	Cases	
	Without AMC	With AMC
Malignant neoplasms and their treatment	1,719 (86.5%)	269 (13.5%)
Chronic diseases of the heart and the lungs	1,071 (87.7%)	150 (12.3%)
Psychosomatic principal diagnoses	164 (80.0%)	41 (20.0%)
Surgical procedures and interventions	91 (47.9%)	99 (52.1%)

**Table 3:** Structural and cost data distribution in cases with/without anthroposophic medical complex (AMC) treatment.

	Without AMC	With AMC
Cases		
Hospital 1	10,617 (97.4%)	273 (2.6%)
Hospital 2	9,405 (92.6%)	697 (7.4%)
Hospital 3	1,827 (80.2%)	361 (19.8%)

Total	21,849 (93.9%)	1,331 (6.1%)

Average length of stay		
All (SD)	6.1 (5.2)	19.5 (14.9)
Inlier (SD)	6.2 (4.9)	14.7 (7.9)
German InEK catalog	6.2	10.9
% under average length of stay		
All	55.7%	5.9%
Inlier	50.7%	10.6%
% cases		
Low outlier	15.1%	0.1%
Inlier	76.0%	53.1%
High outlier	6.8%	46.6%
Transferred^3^	2.0%	0.2%
Remuneration of all cases in €		
DRG income^1^ (sd)	2,473 (2,864)	6,405 (12,619)
ZE income^2^ (sd)	66 (417)	111 (635)
Total income (sd)	2,540 (2,956)	6,516 (12,835)
Remuneration inlier in €		
DRG income^1^ (sd)	2,650 (2,779)	5,863 (11,109)
ZE income^2^ (sd)	57 (392)	86 (606)
Total income (sd)	2,707 (2,865)	5,949 (11,344)
Cost in €		
Cost (all) (sd)	2,417 (3,145)	7,992 (11,019)
Cost (inlier) (sd)	2,451 (3,037)	6,724 (9,323)
Cost (inlier)/ZE income	2,394	6,638
Cost (InEK)	2,387	5,244

^1^DRG income: revenues through lump compensation.

^2^ZE income: revenues through additional remuneration.

^3^Cases transferred to or transferred from other hospitals with length of stay shorter than the average of the respective DRG (and therefore subject to deductions).
